# Silver Nanoparticles Decorated UiO-66-NH_2_ Metal-Organic Framework for Combination Therapy in Cancer Treatment

**DOI:** 10.3390/pharmaceutics17040512

**Published:** 2025-04-13

**Authors:** Francesco Ragonese, Letizia Trovarelli, Lorenzo Monarca, Sofia Girolmoni, Flora Ballarino, Ferdinando Costantino, Bernard Fioretti

**Affiliations:** Department of Chemistry, Biology and Biotechnologies, University of Perugia, Via Elce di Sotto 8, 06123 Perugia, Italy; letizia.trovarelli@dottorandi.unipg.it (L.T.); lorenzomonarca.92@gmail.com (L.M.); sofia.girolmoni@collaboratori.unipg.it (S.G.); flora.ballarino@unipg.it (F.B.); ferdinando.costantino@unipg.it (F.C.)

**Keywords:** silver nanoparticles, MOF, UiO-66, cancer, chemotherapy, combination therapy, drug delivery, nanomedicine

## Abstract

**Background**: Nanomedicine has shown significant promise in advancing cancer diagnostics and therapeutics. In particular, nanoparticles (NPs) offer potential for overcoming limitations associated with conventional therapies, such as off-target toxicity and side effects. Among the various NPs, silver nanoparticles (AgNPs) have garnered attention due to their cytotoxic and genotoxic properties in cancer cells. However, despite their potential, the optimization of AgNPs efficacy often necessitates combination strategies with other therapeutic agents. This study explores the potential of AgNPs integrated with Zr-based metal-organic frameworks (MOFs) UiO-66 for drug delivery, to enhance cancer therapy. **Methods**: We decorated amino-terephthalic based UiO-66-NH_2_ with AgNPs and loaded it with the chemotherapeutic agent cisplatin (Cis-Pt) to make the UiO-66-NH_2_@AgNPs@Cis-Pt. A preliminary MTT assay was conducted to evaluate the cytotoxic effects of the nanocomposite on several glioblastoma and other tumour cell lines, including U251, U87, GL261, HeLa, RKO, and HepG2. **Results**: Our results demonstrate that UiO-66-NH_2_@AgNPs@Cis-Pt and its combinations exhibit enhanced cytotoxicity compared to individual components such as AgNPs and Cis-Pt. **Conclusions**: This work offers preliminary insights into the potential of AgNP-functionalized MOFs as effective drug and delivery platforms, particularly in the context of combination therapy for cancer treatment.

## 1. Introduction

Nanomedicine, born from the combination of nanotechnology with medical practices, is an evolving field that can have an increasingly significant impact in several areas of both diagnostic and therapeutic cancer treatment [1]. In particular, the utilization of nanoparticles (NPs) holds promise for enhancing existing therapeutic modalities or for the development of novel ones. The broad range of NPs that can be obtained, combined with their advanced versatility, drug delivery, and cellular targeting capabilities, has the potential to overcome many of the common limitations of conventional therapies based on direct drug application, including off-target toxicity and undesirable side effects [2].

Among the various NPs that have been studied, silver nanoparticles (AgNPs) are attracting increasing interest due to their interesting cytotoxic and genotoxic characteristics towards cancer cells [3]. The biological activity of AgNPs is influenced by a multitude of factors, including but not limited to surface chemistry, size, size distribution, shape, and coating/capping material [4,5]. Their nanometric structure allows them to easily penetrate and accumulate in tumour tissues either actively, via targeting systems [6,7,8], or passively, by taking advantage of tumour angiogenesis and the enhanced permeation and retention (EPR) effect, where the penetration and accumulation of nano-sized materials within tumoral mass is facilitated by the combination of atypical endothelial layers and fenestrated vasculature with impaired lymphatic drainage [9,10,11]. When in contact with tumour cells, AgNPs can be internalised by passive diffusion or endocytosis depending on their size. Once inside, AgNPs induce an increase in the production of reactive oxygen species (ROS) and cause mitochondrial dysfunctions, caspases activation, apoptosis, autophagy, and DNA damage [12]. Due to these characteristics, numerous works have evaluated their potential therapeutic use against various forms of cancer in both in vitro [8,13,14,15] and in vivo [16,17,18,19] models.

Despite remarkable anti-cancer effect of AgNPs, the achievement of optimal efficacy may require the combination of these agents with other anti-cancer drugs to target the diverse vulnerabilities of cancer cells. Fahrenholtz et al. investigated the effects of AgNPs exposure in ovarian cancer cell lines (A2780, SKOV3, and OVCAR3) in combination with cisplatin (Cis-Pt), demonstrating a synergistic effect on cellular cytotoxicity [20]. Another study examines the role of heat shock response and the integrated stress response in AgNP-induced cytotoxicity in triple-negative breast cancer (TNBC) and immortalized mammary epithelial cells. The combination of AgNPs and salubrinal increases the anti-cancer efficacy of AgNPs against TNBC without increasing off-target toxicity in immortalized mammary epithelial cells [21]. Furthermore, a strong synergistic effect was observed between the cellular toxicity of AgNPs and various histone deacetylase inhibitors on the HeLa cervical, A549 lung cancer cell lines [22,23].

Given the advantages that combination therapies can offer, it is worthwhile to develop methods to optimize the delivery of AgNPs and chemotherapeutics. One potential approach involves the combination of AgNPs with nano-structured drug delivery systems, which have the capacity to internalise drugs and release them concomitantly within cancer cells. Among the various classes of nanomaterials suitable for this purpose, metal-organic frameworks (MOFs) may be perfect candidates due to their porosity, versatility, and biocompatibility. MOFs are porous organic–inorganic coordinated materials synthesised through self-assembly reactions, where metal ions form cluster nodes that bond with organic linker molecules as ligands, forming a porous crystalline nanostructure [24]. The primary focus of research on MOFs is their application in the field of drug delivery due to their unique properties [25]. These features include controllable morphologies, adjustable diameters, varied compositions, tuneable porosities, high specific surface areas, ease of functionalisation, and favourable physicochemical properties [26]. MOFs can be used in cancer therapy in both monotherapies and combination strategies [27,28]. In this field, we previously reported the synthesis and characterisation of ultrasmall UiO-66 based on a [Zr_6_O_4_(OH)_4_]^12+^ hexanuclear cluster combined with 1,4-benzodicarboxylic acid. The internalisation of the MOF nanoparticles, without any surface modification, was observed in U251 human glioblastoma cells. The NPs were retained within the cells without inducing morphological changes or decreasing cell viability [29]. In the present work, we decorated UiO-66-NH_2_ with AgNPs and loaded the construct with the chemotherapeutic drug cisplatin (Cis-Pt). The amine group of aminoterephthalic acid, used as a ligand, can promote strong metal interaction between the available amine groups and AgNPs to achieve effective Ag loading [30]. We then tested its cytotoxic efficacy against several human and murine glioblastoma tumour lines (U251, U87 and GL261) and three other tumour lines (HeLa, RKO, and HepG2) in order to verify the enhancement efficacy of the combined effect of AgNPs decorated UiO-66-NH_2_ and Cis-Pt.

## 2. Materials and Methods

### 2.1. Reagents and Synthesis of Zr-NH_2_BDC (UiO-66-NH_2_)

Except where otherwise specified, all reagents come from Sigma-Aldrich (Merck, Darmstadt, Germany). A minor adjustment to the established literature procedure was implemented for the synthesis of Zr-NH_2_ BDC (UiO-66-NH_2_) [31]. The synthesis was carried out by adding ZrCl_4_ (1.71 mmol) to 75 mL of dimethylformamide (DMF) and subsequently adding 2.85 mL of acetic acid. Subsequently, the NH_2_ BDC was incorporated into the mixture, dissolved in 25 mL of DMF, and then transferred to the preceding solution. Subsequently, 0.125 mL of water was added. The reaction was then conducted under reflux (120 °C) for 24 h. The solid was then recovered by centrifugation and washed twice in acetone and once in water. The product was then dried at 60 °C for 12 h.

### 2.2. Decoration of AgNPs on UiO-66-NH_2_

A total of 200 mg of UiO-66-NH_2_ was dispersed in 30 mL of water upon ultrasonication for 15 min. Then, 4 mL of AgNO_3_ 0.005 M was added. The mixture was stirred at room temperature for 24 h. In order to reduce Ag^+^ to metallic Ag nanoparticles, 2.4 mL of NaBH_4_ (0.4 M) was introduced and the dispersion was stirred for 30 min. The formation of AgNPs resulted in a colour change from yellow to brown. The solid was recovered by centrifugation and washed three times in water and once in acetone. The product was dried overnight at 60 °C [32,33].

### 2.3. Cis-Pt Loading Procedure in UiO-66-NH_2_

The Cis-Pt loading procedure was adapted from the literature [34]: 50 mg of UiO-66-NH_2_ was dispersed in 5 mL of Cis-Pt solution (0.05 M) in dimethyl sulfoxide (DMSO). The solution was stirred for three days and then recovered through centrifugation. The solid was washed three times in methanol and then dried at 60 °C for 12 h.

### 2.4. Inductively Coupled Plasma Optical Emission Spectroscopy (ICP-OES)

All samples were dissolved by using a 3% nitric acid solution for 24 h until complete dissolution of the materials occurred. To quantify the Pt and Ag content in the UiO-66- NH_2_, an ICP-OES analysis was performed. A Perkin Elmer spectrometer (Waltham, MA, USA) was used for ICP-OES analysis.

### 2.5. Scanning Electron Microscopes (SEM) Analysis

All samples were deposited in the stub and then sputter-coated under vacuum with chromium to make the sample conductive. Field emission scanning electron microscopy (FE-SEM) images were obtained using a LEO 1525 Gemini SEM (Zeiss/LEO, Jena, Germany) with an acceleration voltage of 5.00 kV (USA).

### 2.6. Powder X-Ray Diffraction

Powder X-ray diffraction (PXRD) patterns were collected with a Bruker diffractometer D8 ADVANCE (Billerica, MA, USA) with a Cu-Kα1,2 anode (λ = 1.5406 Å). Mercury software 2023.2.0 was used for data visualization.

### 2.7. Brunauer–Emmett–Telle (BET) Analysis

A Micromeritics 2010 and a Gemini VII Micromeritics analysers (Micromeritics, Norcross, GA, USA) were used to obtain the adsorption and desorption isotherms with nitrogen at 77 K to measure the Brunauer−Emmett−Teller (BET) surface. Before the adsorption analysis, the samples were outgassed at 100 °C under vacuum overnight before starting the measurement.

### 2.8. Dynamic Light Scattering (DLS)

Dynamic light scattering (DLS) analysis was performed with a Litesizer DLS 500 instrument (Graz, Austria). The samples were dispersed in water and the suspensions were then sonicated for 10 min to prepare the colloidal solution and for another 10 min before the DLS analysis.

### 2.9. Cell Cultures

U251 cells were obtained from Cell Lines Service (CLS), GmbH Culture Collection (Eppelheim, Germany), U87 and HepG2 cells from American Type Culture Collection ATCC (Manassas, YA, USA), and GL261 cells from DMSZ (Leibniz Institute, Jena, Germany). The HeLa and RKO cell lines were kindly donated by Professor Giuseppe Servillo of the Department of Medicine and Surgery at the University of Perugia. All cell lines were cultivated in phenol-red Dulbecco’s Modified Eagle Medium (DMEM) with 10% fetal bovine serum, 100 IU/mL penicillin/streptomycin, and 200 mM L-glutamine. The cells were grown in 25 cm^2^ flasks (Falcon, Corning, Glendale, AZ, USA). The flasks were then placed in an incubator at 37 °C in a 5% CO_2_ humidified atmosphere. The medium was changed twice a week, and the cells were subcultured with Trypsin/EDTA 0.025% when they reached 80% of confluence. All reagents and media used for cell culture maintenance were purchased from Euroclone, Pero, IT.

### 2.10. MTT Viability Assay

To assess the potential toxicity of the constructs under investigation, the MTT assay was used to determine cell viability and proliferation. In order to ensure the optimal growth of cells, 3.5 × 10^3^ cells/well were plated with complete medium in a 96-well plate for the U251 and GL261 cell lines, whereas 5 × 10^3^ cells/well were plated for the U87, HepG2, HeLa, and RKO cell lines. After 24 h of incubation, the cells were exposed to different concentrations of UiO-66-NH_2_@AgNPs (0.1, 0.5, 1, 5, and 10 μg/mL) or to different combinations of UiO-66-NH_2_ constructs (UiO-66-NH_2_, UiO-66-NH_2_@AgNPs, UiO-66-NH_2_@Cis-Pt, and UiO-66-NH_2_@AgNPs@Cis-Pt). The MOFs were resuspended in water and sonicated briefly prior to being added directly to the culture medium. AgNPs at 0.009 μg/mL and Cis-Pt at 0.325 μg/mL were used as single-component controls. After 24 and 48 h of treatment, the cells were incubated with 0.5 mg/mL 3-(4,5-dimethylthiazol-2-yl)-2,5-diphenyltetrazolium bromide (MTT) (Sigma-Aldrich, Merck, Darmstadt, Germany) for 3 h. Following this incubation, the medium was removed, and 200 µL of Dimethyl Sulfoxide (DMSO) (Sigma-Aldrich, Merck, Darmstadt, Germany) was added to the wells. The analysis of cell viability was conducted at a wavelength of 550 nm using the Infinite M Nano+ (Tecan Trading AG, Männedorf, Switzerland). The results were expressed as a normalized percentage based on the ratio of the absorbance of treated cells to that of untreated controls.

### 2.11. Statistical Analysis

All experiments were performed at least three times independently. Data are expressed as the mean ± standard deviation (SD). Data were analysed using a one-way ANOVA test in combination with Dunnett’s multiple comparison test. *p* < 0.05 (*), *p* < 0.01 (**), *p* < 0.001 (***), and *p* < 0.0001 (****) were used to assess the significance of the results. All statistical analyses were performed using Prism Graph Pad 9 software and Origin Lab 6.5 software.

## 3. Results

### 3.1. Synthesis and Characterisation of AgNPs Decorated UiO-66-NH_2_ Loaded with Cis-Pt

#### 3.1.1. Synthesis of UiO-66-NH_2_

**Figure 1:**
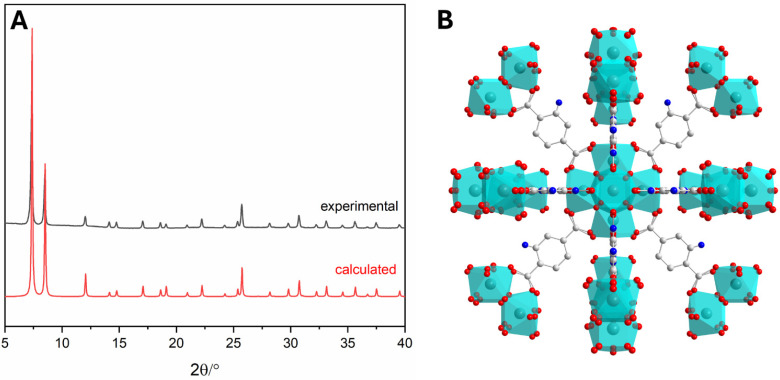
(**A**) PXRD patterns of UiO-66-NH_2_ as-synthetised material (black) and the calculated one (red) and UiO-66-NH_2_ crystal viewed along c-axis. (**B**) Colour scheme of UiO-66-NH_2_: Zr in light blue, O in red, N in blue, and C in grey (hydrogen atoms are omitted for clarity).

The synthesis was successfully confirmed by X-ray powder diffraction (PXRD) analysis (Figure 1A): all the peaks, especially the most evident at 7.3°, 8.5°, and 12° of 2-theta, were coincident with the reported values in the literature for UiO-66-NH_2_ [35].

#### 3.1.2. Decoration of UiO-66-NH_2_ with AgNPs

**Figure 2:**
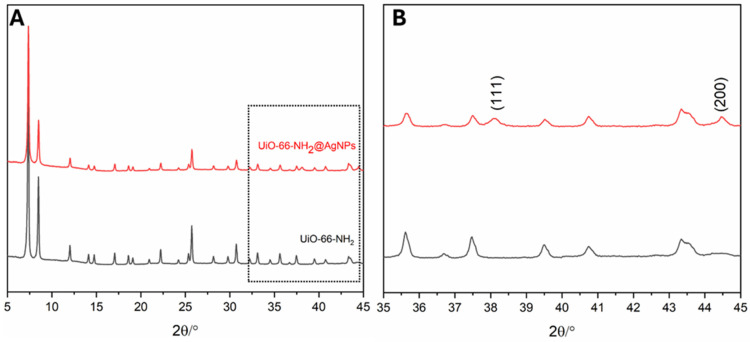
PXRD patterns of (**A**) UiO-66-NH_2_ as-synthetized (black) and UiO-66-NH_2_ decorated with Ag metallic (red) and (**B**) enlargement of figure highlighting the reflections of metallic AgNPs.

The PXRD patterns of the as-prepared UiO-66-NH_2_ and UiO-66-NH_2_ decorated with AgNPs are depicted in Figure 2. The patterns of UiO-66-NH_2_@AgNPs are in good agreement with the results reported earlier. In the UiO-66-NH_2_@AgNPs sample (Figure 2B), two new peaks at ca. 38.2° and 44.5° of 2-theta, corresponding to the hkl reflections of (111) and (200) of metallic silver, appeared. This suggests that decoration of the cubic structure with AgNPs occurred [36]. The crystallite size of the AgNPs, calculated by using the Scherrer equation considering the two reflections, is around 30 nm.

**Figure 3:**
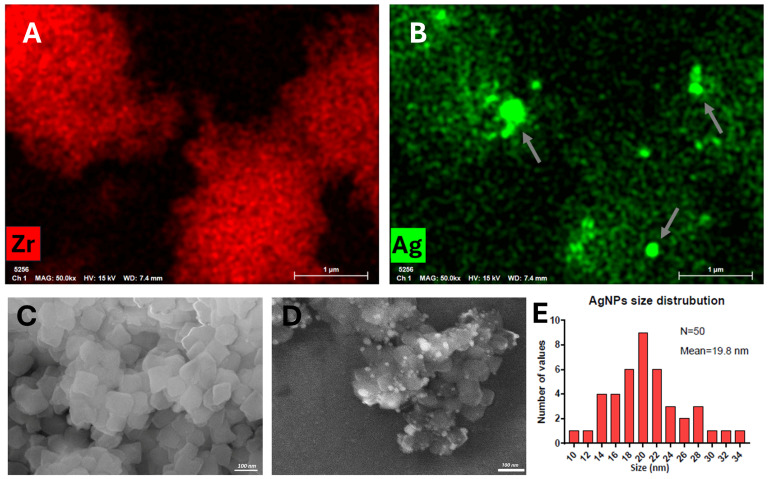
EDX images of UiO-66-NH_2_@AgNPs highlight the presence of Zr (**A**) and the aggregates of AgNPs indicated by the white arrows (**B**). (**C**) SEM images of UiO-66-NH_2_ as-synthetized. (**D**) Secondary electrons SEM image showing the presence of AgNPs on UiO-66-NH_2_ surface (white sphere). (**E**) Graph illustrates the size distribution of AgNPs.

EDX images depicted in Figure 3A,B and in the Supplementary Figure S1, confirmed the presence of both Zr and Ag within the material. As shown in Figure 3D, the secondary electron microscopy (SEM) images reveal that the UiO-66-NH_2_ particles are decorated with metallic AgNPs. In the SEM images (Figure 3C,D), UiO-66-NH_2_ appears as symmetrical crystals with triangular base pyramid morphology. The size of UiO-66-NH_2_ was determined to be approximately 100 nm. Similarly, the size of AgNPs is distributed around the value of 20 nm, as depicted in Figure 3E and confirmed by the SEM images in Figure 3D. The size obtained using the Scherrer equation is different because nanoparticles tend to form aggregates, as evidenced in Figure 3B.

#### 3.1.3. Loading UiO-66-NH_2_ with Cis-Pt

**Figure 4:**
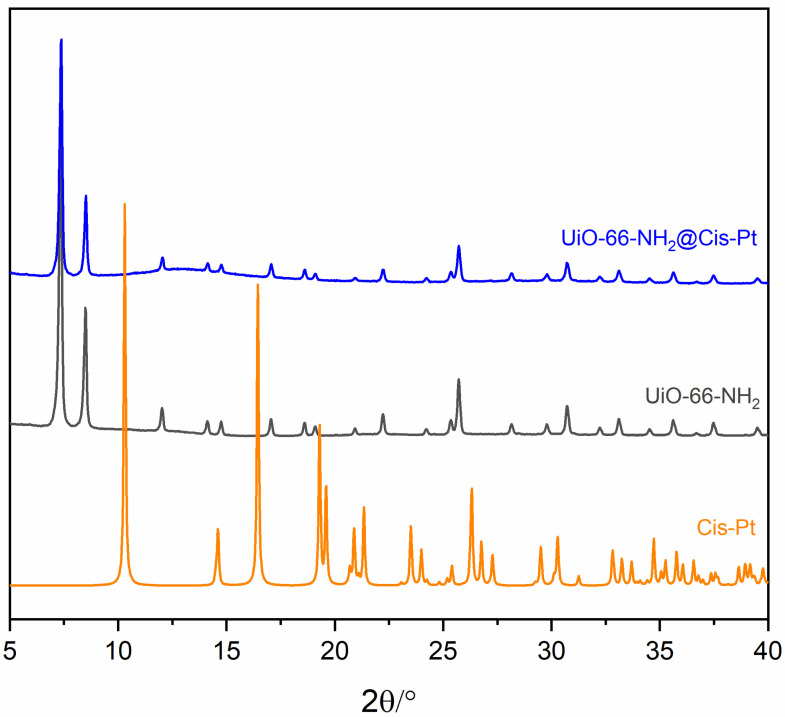
PXRD patterns of UiO-66-NH_2_ as-synthetized (black) and UiO-66-NH_2_ loaded with Cis-Pt (blue) and the calculated pattern of Cis-Pt (orange).

The PXRD patterns reported in Figure 4 show that the Cis-Pt loading procedure does not affect the stability and crystallinity of the UiO-66-NH_2_ framework. The reflections of Cis-Pt (orange line) are not present in the PXRD pattern of UiO-66-NH_2_@Cis-Pt (Figure 2A), indicating the absence of two separate phases between the MOF and Cis-Pt. The effective presence of Cis-Pt is confirmed from the EDX spectra reported in Figure S2 and from the ICP-OES analysis data reported in Table 1.

#### 3.1.4. Loading UiO-66-NH_2_@AgNP with Cis-Pt

**Figure 5:**
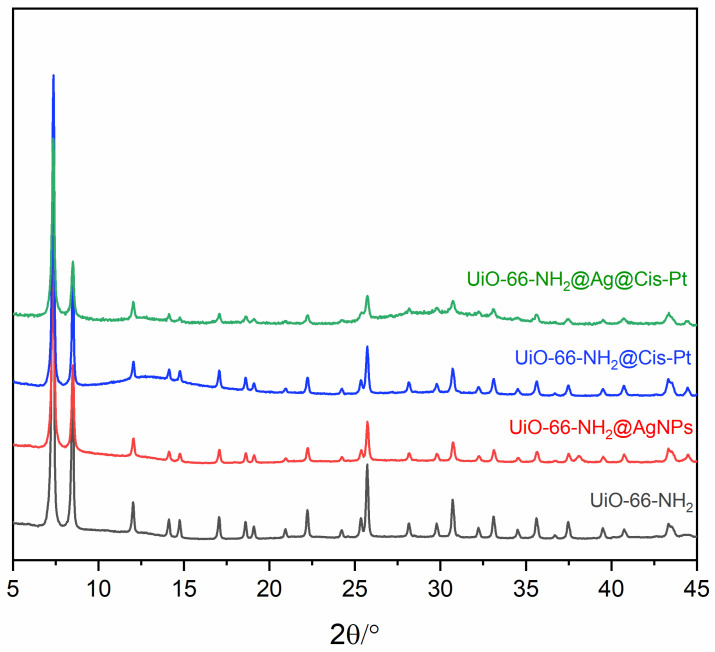
PXRD patterns of UiO-66-NH_2_ as-synthetized (black), UiO-66-NH_2_ decorated AgNPs (red), UiO-66-NH_2_ loaded with Cis-Pt (blue), and UiO-66-NH_2_ loaded with both AgNPs and Cis-Pt (green).

The PXRD patterns of all the materials are reported in Figure 5. The double treatment, consisting of environmental reduction to allow AgNPs decoration and the subsequent Cis-Pt loading in DMSO, does not affect the structure of the material, highlighting the characteristic stability of Zr-based MOFs like UiO-66-NH_2_ [37].

#### 3.1.5. BET Surface Area and ICP-OES Quantification

BET analysis (reported in Figures S3 and S4) shows that the presence of AgNPs reduces the specific surface area and pore volume of UiO-66-NH_2_ from 1037 m^2^/g to 805 m^2^/g, respectively. These results indicate that the presence of AgNPs in our system still maintains the loading capacity of UiO-66-NH_2_ crystals. The presence of Cis-Pt was also confirmed by the BET analysis. In fact, the micropore surface area decreased from 1037 m^2^/g of the pristine material to 931 m^2^/g. The surface area also decreased when the AgNPs decorated UiO-66-NH_2_ was loaded with Cis-Pt, confirming its presence in the pores but still ensuring its loading capacity. The ICP-OES data were employed in order to proceed with the following procedure for the determination of controls concentration.

#### 3.1.6. Dynamic Light Scattering (DLS) Analysis

DLS analysis was performed by dissolving the samples in deionized water at two different concentrations: 10 µg/mL and 0.5 mg/mL. The concentration of 10 µg/mL was selected based on its equivalence to the concentration employed in the subsequent experiment conducted on cell lines. However, the DLS results when using the concentration of 10 µg/mL did not provide any substantial information on the particle size, as the correlation function showed a noisy baseline. In that case, the instrument counts were insufficient to provide a statistically significant estimate of the particle size in suspension. For this reason, DLS data from the more concentrated solution (0.5 mg/mL) were considered, as they exhibited a correlation function with a flatter baseline (Figures S5–S8).

As shown in Figure S5, the DLS analysis of UiO-66-NH_2_ confirms the dimensional distribution observed in the SEM images, with a hydrodynamic diameter of 243.4 nm. In contrast, the other composites exhibit coalescence phenomena, leading to the formation of aggregates smaller than 2.5 µm. This can be explained by the fact that the MOF surface was modified during the synthesis procedure through the addition of AgNPs and Cis-Pt, making it less hydrophilic.

Additionally, in the case of UiO-66-NH_2_@AgNPs (Figure S6) and UiO-66-NH_2_@AgNPs@Cis-Pt (Figure S8), another particle population was observed, centred at 166.34 nm (with an area of 22.98%) and 131.07 nm (with an area of 2.03%), respectively. To explain this behaviour in suspension, certain hypotheses can be proposed, including the release of nanoparticles from the MOF surface and the subsequent possible aggregation of the AgNPs. Concerning UiO-66-NH_2_@Cis-Pt (Figure S7), only one population was detected, with a hydrodynamic diameter of approximately 1.75 µm, larger than that of UiO-66-NH_2_. The SEM analysis and ICP-OES data obtained in the preceding experiments support this consideration.

### 3.2. Toxicity Effects of UiO-66-NH_2_@AgNPs@Cis-Pt on Cancer Cell Lines

#### 3.2.1. UiO-66-NH_2_@AgNPs Reduce Vitality of Cells in Dose-Dependent Manner

**Figure 6:**
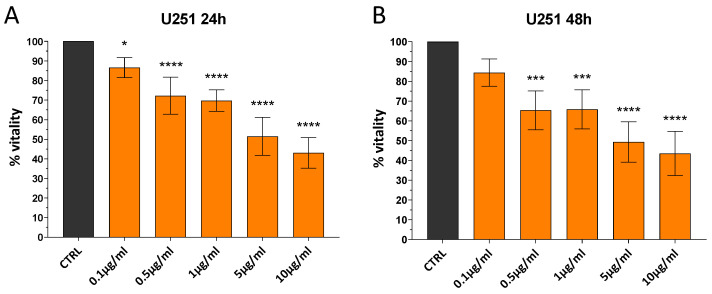
Dose-dependent response of U251 to UiO-66-NH_2_@AgNPs. Bar plot of MTT assay of U251 glioblastoma cell line incubated with 0.1, 0.5, 1, 5, and 10 μg/mL UiO-66-NH_2_@AgNPs for 24 h (**A**) and 48 h (**B**). Cells without treatments were used as controls. The bars represent the mean values of at least three experiments plus SD. * *p* < 0.05, *** *p* < 0.001, and **** *p* < 0.0001 indicate the significance obtained with Dunnett’s multiple comparison test of treated cells vs. untreated control cells.

In order to determine the appropriate concentration of AgNP@UiO-66-NH_2_ for cell treatment, a dose-response curve was generated for the application of UiO-66-NH_2_@AgNPs (0.1, 0.5, 1, 5, and 10 μg/mL) to the U251 human glioblastoma cell line, and the MTT viability assay was used to evaluate the effects of treatment. As shown in Figure 6A,B, UiO-66-NH_2_@AgNPs affect cell viability at all concentrations tested. In particular, about 50% of viability reduction was detected after both 24 and 48 h in cells treated with 5 µg/mL of UiO-66-NH_2_@AgNPs. This concentration was the optimal condition for observing the eventual enhancement and decrease in the biological effects of our constructs. Therefore, we applied 5 µg/mL of UiO-66-NH_2_@AgNPs in the subsequent experiments.

#### 3.2.2. Effect of UiO-66-NH_2_@AgNPs on Glioblastoma Cell Lines

**Figure 7:**
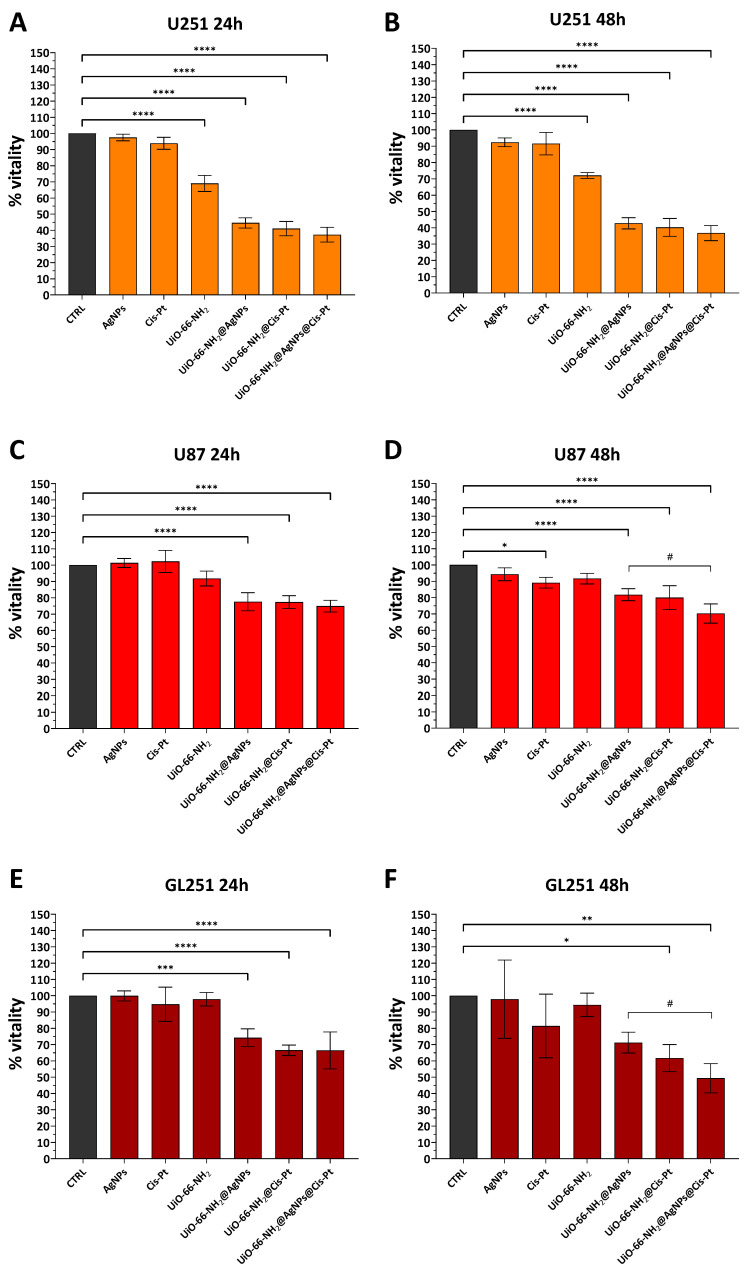
Effect of decorated UiO-66-NH_2_ on three different glioblastoma cell lines. Bar plot of MTT assay of U251 (**A**,**B**), U87 (**C**,**D**), and GL261 (**E**,**F**) glioma cell lines incubated with 5 μg/mL of UiO-66-NH_2_, UiO-66-NH_2_@AgNPs, UiO-66-NH_2_@Cis-Pt, and UiO-66-NH_2_@AgNPs@Cis-Pt for 24 and 48 h. Cells without treatments were used as controls, while 0.009 μg/mL AgNPs, 0.325 μg/mL Cis-Pt and 5 μg/mL of UiO-66-NH_2_ were used to evaluate the effects of the individual components. The bars represent the mean values of at least three experiments plus SD. * *p* < 0.05, ** *p* < 0.01, *** *p* < 0.001, and **** *p* < 0.0001 indicate significance obtained using Dunnett’s multiple comparison test of treated cells vs. untreated control cells. # *p* < 0.05 indicate the significance obtained using Dunnett’s multiple comparison test of UIO-66-NH_2_@AgNPs@Cis-Pt vs. UiO-66-NH_2_@AgNPs and UiO-66-NH_2_@Cis-Pt.

For the evaluation of the cytotoxic effects of UiO-66-NH_2_@AgNPs and possible synergistic effects with conventional anticancer agents, three different glioblastoma lines (human U251 and U87, and murine GL251) were treated with 5 μg/mL UiO-66-NH_2_@AgNPs, UiO-66-NH_2_@Cis-Pt, and UiO-66-NH_2_@AgNPs@Cis-Pt. The individual components (UiO-66-NH_2_ alone, AgNPs, and Cis-Pt) were used as controls. UiO-66-NH_2_ were tested at the concentration of 5 μg/mL, while the final concentrations of AgNPs and Cis-Pt were determined based on the data obtained from ICP (0.009 μg/mL and 0.325 μg/mL, respectively). The results show that MOFs decorated with AgNPs or loaded with Cis-Pt have a significant cytotoxic effect compared to that of the individual components (Figure 7). This effect is most pronounced in the case of U251, where a reduction of approximately 55% for UiO-66-NH_2_@AgNPs and 59% for UiO-66-NH_2_@Cis-Pt is observed at 24 h, and it remains almost constant at 48 h (Figure 7A,B). In contrast, less pronounced effects are observed in U87 and GL261, where the same treatments only reduce viability values by about 23.5% and 23.2% for U87 and by 26% and 34% for GL261, 24 h after treatment (Figure 7C,E). As previously observed for U251, these values do not change significantly at 48h (Figure 7D,F). UiO-66-NH_2_@AgNPs@Cis-Pt exhibited a marginal additive combined effect of AgNPs and Cis-Pt on MTT values compared to UiO-66-NH_2_@AgNPs and UiO-66-NH_2_@Cis-Pt at all time points analysed. However, the difference was not significant except in the case of GL261 treated for 48 h, where a variance of approximately 22% between UiO-66-NH_2_@AgNPs and UiO-66-NH_2_@AgNPs@Cis-Pt was observed (Figure 7F).

#### 3.2.3. Effect of UiO-66-NH_2_@AgNPs on Various Tumour Cell Lines

**Figure 8:**
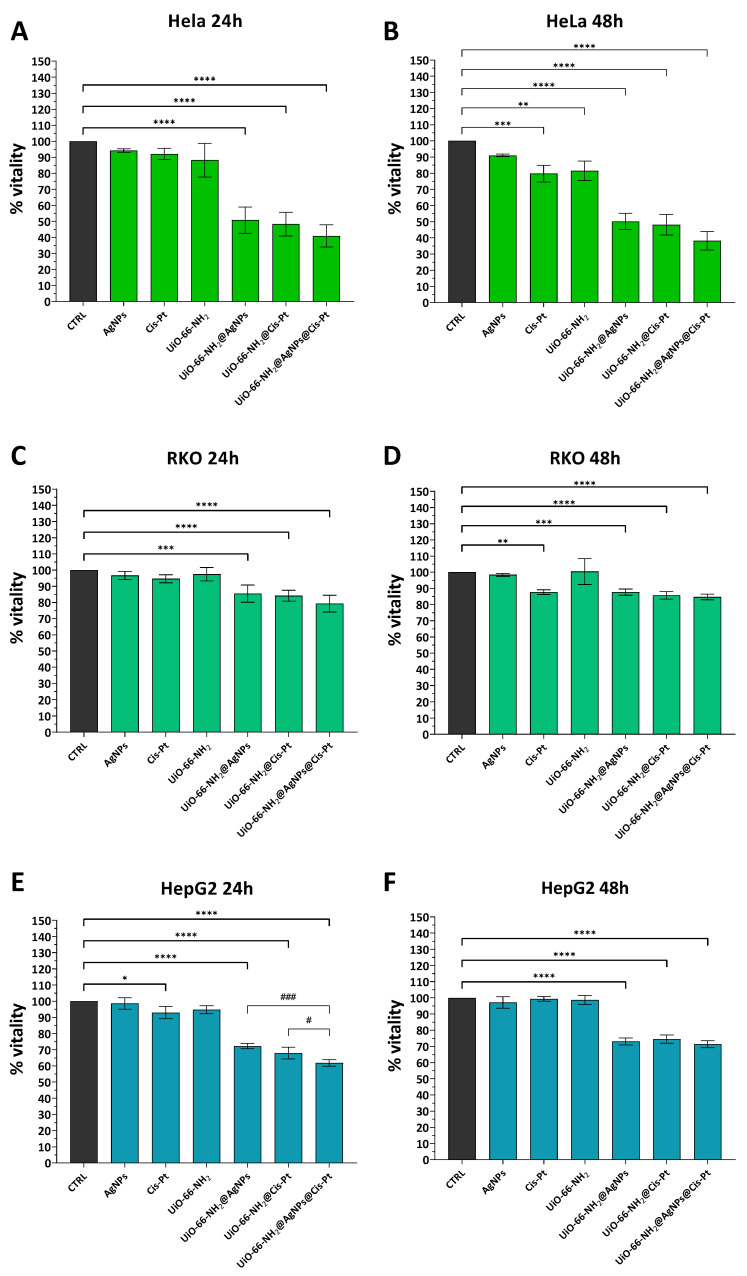
Effect of decorated UiO-66-NH_2_ on three different cancer cell lines. Bar plot of MTT assay of HeLa (**A**,**B**), RKO (**C**,**D**) and HepG2 (**E**,**F**) cell lines incubated with 5 μg/mL of UiO-66-NH_2_, UiO-66-NH_2_@AgNPs, UiO-66-NH_2_@Cis-Pt and UiO-66-NH_2_@AgNPs@Cis-Pt for 24 and 48 h. Cells without treatments were used as controls, while 0.009 μg/mL AgNPs, 0.325 μg/mL Cis-Pt and 5 μg/mL of UiO-66-NH_2_ were used to evaluate the effects of the individual components. The bars represent the mean values of at least three experiments plus SD. * *p* < 0.05, ** *p* < 0.01, *** *p* < 0.001, and **** *p* < 0.0001 indicate the significance obtained using Dunnett’s multiple comparison test of treated cells vs. untreated control cells. # *p* < 0.05 and ### *p* < 0.001, indicate the significance obtained using Dunnett’s multiple comparison test of UIO-66-NH_2_@AgNPs@Cis-Pt vs. UiO-66-NH_2_@AgNPs and UiO-66-NH_2_@Cis-Pt.

In order to assess the cytotoxic efficacy of our constructs on tumour cell lines other than glioblastoma, the same MTT assays were performed on uterine carcinoma (HeLa), colon epithelial carcinoma (RKO), and liver cancer (HepG2) cell lines. The results obtained from these experiments were consistent with those previously observed in glioma cells. Data shows that the UiO-66-NH_2_@AgNPs and UiO-66-NH_2_@Cis-Pt combinations have a greater effect than the individual components at both time points tested (Figure 8). The greatest effect is observed in HeLa. At 24 h, the reduction values are approximately 49.2% for UiO-66-NH_2_@AgNPs and 51.2% for UiO-66-NH^2^@Cis-Pt. At 48 h, similar values of about 49% and 52.1%, respectively, are obtained (Figure 8A,B). The situation is similar for HepG2 cells. A decline of nearly 28% and 32% was observed at 24 h, and 27% and 25.5% at 48 h when UiO-66-NH_2_@AgNPs and UiO-66-NH_2_@Cis-Pt were administered. In RKOs, the reduction in viability is less pronounced and is around 14.6% for UiO-66-NH_2_@AgNPs and 16% for UiO-66-NH_2_@Cis-Pt at 24 h, values that do not change significantly at the following 24 h (13.3% and 14.4%, respectively, Figure 8C,D). The UiO-66-NH_2_@AgNPs@Cis-Pt combination does not significantly alter viability in Hela, although there seems to be a slight additive effect (Figure 8A,B). No significant changes were observed in the RKOs when comparing the values of UiO-66-NH_2_@AgNPs and UiO-66-NH_2_@Cis-Pt with the values of UiO-66-NH_2_@AgNPs@Cis-Pt (Figure 8C,D). In the case of HepG2, a significant reduction in vitality was observed in relation to both UiO-66-NH_2_@AgNPs (approximately 11%) and UiO-66-NH_2_@Cis-Pt (about 7%) after 24 h of treatment with UiO-66-NH_2_@AgNPs@Cis-Pt (Figure 8E,F).

## 4. Discussion

Chemotherapy in cancer is still ineffective in many cases due to a number of limiting factors such as toxicity, drug resistance, and the ability of cancer cells to bypass the toxic effects of the substances. One way to make treatments more effective is to apply combination treatments, in which two or more drugs are administered simultaneously in order to target different features of the cancer cells. From this perspective, nanomedicine, and particularly the use of nanoparticles, represent a promising and evolving field [38,39].

In this work, we tried to combine the antitumour properties of AgNPs with the transport capabilities of the MOFs UiO-66-NH_2_, to create a nano-construct capable of simultaneously applying AgNPs and a chemotherapeutic drug. We have synthesised a well-established Zr-based MOF UiO-66 containing aminoterephthalic acid as a linker. The presence of a porous MOF nanostructure with a size of approximately 100 nm, supported by Ag nanoclusters ranging from 10 to 20 nm, has been confirmed through PXRD, BET analysis, and SEM imaging. Given the evidence from several studies demonstrating the enhanced efficacy of the combination of AgNPs and Cis-Pt compared to the individual components in various tumour models [40,41,42,43], the latter substance was selected as the chemotherapeutic agent to be incorporated into our UiO-66-NH_2_@AgNPs@Cis-Pt. The size of Cis-Pt allowed us to easily incorporate it into the UiO-66-NH_2_ porous cages, and the results obtained from PXRD, ICP-OES, and BET surface area analyses showed the effective decoration and loading of both AgNPs and Cis-Pt.

The UiO-66-NH_2_@AgNPs@Cis-Pt and its combinations—UiO-66-NH_2_@AgNPs and UiO-66-NH_2_@Cis-Pt—were tested using the MTT assay to determine the viability of three distinct glioblastoma cell lines (U251, U87, and GL261) and three additional tumour cell lines (HeLa, RKO, and HepG2) at 24 and 48 h. The cytotoxic effects were evaluated indirectly by measuring the quantity of MTT converted into formazan crystals by mitochondrial enzyme succinate dehydrogenase. The assumption is that, under saturating conditions, the quantity of formazan produced is proportional to the number of cells. The results were compared with those obtained from the individual components (UiO-66-NH_2_, AgNPs, and Cis-Pt). In most cases, 5 μg/mL of UiO-66-NH_2_ alone seems to be biocompatible and does not reduce the cell vitality significantly, except in the case of U251 or Hela at 48 h. In particular, U251 appears to be particularly sensitive to UiO-66-NH_2_. Since we have already demonstrated the biocompatibility of UiO-66 and other Zirconium- or Zinc-based MOFs [29,44], it seems the increased sensitivity of these cells is attributed to the addition of the amine group to the UiO-66 shell. AgNPs alone do not seem to be able to give significant effects on viability in any of the cell lines tested, and Cis-Pt causes only a slight reduction in MTT in HepG2 at 24 h and in HeLa and RKO at 48 h. Conversely, UiO-66-NH_2_@AgNPs, UiO-66-NH_2_@Cis-Pt, and UiO-66-NH_2_@AgNPs@Cis-Pt show a significantly increased cytotoxic effect when compared to the individual components. The concentration of AgNPs and Cis-Pt in the constructs, estimated by ICP-OES, is comparable to that of the controls. This suggests that the two components may exert a greater biological effect in combination with UiO-66-NH_2_ than if they were used separately. In the case of AgNPs, this phenomenon may be attributed to the formation of a protein corona. The protein corona, also known as the biomolecular corona, is a layer of amino acids, proteins, and organic molecules present in biological fluids that adsorb onto the surface of the nanoparticle. This phenomenon is important because it has the capacity to modify the properties of NPs [45,46]. One of the primary characteristics of AgNPs is their ability to release Ag+ ions from their surface in response to low pH or oxidation. It has been established that Ag^+^ ions are the principal agents responsible for the cytotoxic effects of AgNPs. These effects include, but are not limited to, DNA damages, increased ROS production, necrosis, and apoptosis [47,48,49]. The protein corona layer that is formed can become large or bulky enough to cover the surface of the AgNP and its functional groups. This can render the NP less effective or modify its ability to internalise within cells [50]. In this case, the presence of MOFs would act as a shield, thereby preventing the excessive formation of protein corona on AgNPs due to electrostatic effects or stearic hinderance. In addition, the presence of MOFs scaffold can facilitate the internalization of AgNPs within the cells. Once in contact with the cell, UiO-66 are able to penetrate inside the cell via a clathrin-mediated endocytosis pathway [51]. Also, it has been observed that the presence of human serum albumin may facilitate the uptake of UiO-66 within Hela cells [52]. In the case of Cis-Pt, its low level of cytotoxic activity when administered alone could in part be due to its degradation by the medium. It is known that Cis-Pt is capable of reacting with numerous species in biological fluids to form fixed and mobile metabolites. The chloride ion concentration and pH were a major determinant of decomposition. Also, albumin could play a significant role in the decomposition of Cis-Pt in biological fluids such as plasma [53]. UiO-66-NH_2_, by internalizing Cis-Pt, can protect it from the degrading effects of the culture media prior to internalization. Clathrin-mediated endocytosis of the UiO-66-NH_2_@Cis-Pt allows the NP to reach acidic compartments, such as lysosome, where the MOFs scaffold can be degraded [54]. Once released form the scaffold, Cis-Pt can diffuse trough the membrane and reach various cellular compartments or cytoplasm, increasing its bioavailability and cytotoxic effects.

Despite the remarkable cytotoxic effect of the UiO-66-NH_2_@AgNPs and UiO-66-NH_2_@Cis-Pt combinations, which is greater than that of the respective individual components, the additive effect of AgNPs and Cis-Pt observed in the case of UiO-66-NH_2_@AgNPs@Cis-Pt compared to the latter, was not so pronounced. It is known that both AgNPs and Cis-Pt lead to DNA damage with the consequence of inducing a cell cycle block, which can evolve into apoptosis [4,55]. In our case, the concentration of the two elements may not be sufficiently high to induce an apoptotic effect. It is likely that the DNA repair mechanism is capable of compensating for most of the damage during the cell cycle arrest, even in the presence of both compounds. This can also explain the fact that no significant increase in toxicity is observed for UiO-66-NH_2_@AgNPs@Cis-Pt or the other construct between 24 and 48 h. As shown in the results, MTT values do not change significantly between the two time points, indicating that the observed effect is more likely to be of a cytostatic nature rather than a cytotoxic nature.

This is certainly a preliminary observation that will have to be further investigated. Additional analyses to verify the state of the cell’s cycle, the number of apoptotic cells, the pathways involved, and the biocompatibility test on non-cancer cells will have to be conducted. Also, the fact that these particles can concentrate in lysosomes may turn into a limitation for non-metal-based drugs. It is known that Cis-Pt is resistant to the acidic environment of the lysosome. AgNPs can undergo degradation in the same location by low pH, thereby releasing Ag^+^. Both can cross biological membranes by diffusion and perform their cytotoxic functions [56,57]. However, other non-metal PH-sensitive drugs may be degraded or excreted before they can have any effect. In this case, further functionalisation of UiO-66-NH_2_ scaffold will be required to prevent accumulation in lysosomes.

However, despite significant room for improvement, the results demonstrate that these nanoparticles exhibit enhanced efficacy in delivering drugs and AgNPs at very low doses.

In our experiments, at equivalent concentrations, the individual components (0.009 μg/mL of AgNPs and 0.325 μg/mL of Cis-Pt) fail to elicit an effective response in the tumour cells examined, whereas when delivered by UiO-66-NH_2_, they exhibit a considerably augmented effect. According to the literature, the concentration of Cis-Pt required to reduce the viability of cancer cells by 50% (IC50) after 48 h averages between 1 and 5 µg/mL [58,59,60]. For AgNPs, the required concentration ranges from 1.5 to 25 µg/mL, depending on the cell type, coating, diameter, and the synthesis method used [14,61,62,63]. This finding suggests that our nanoparticle construct possesses the potential to deliver chemotherapeutics at lower concentrations than necessary, thereby reducing the toxicity and side effects of treatments. Thus, UiO-66-NH_2_@AgNPs@Cis-Pt appears to be a promising model for introducing an efficient delivery system that could prove useful for combination cancer therapy with NPs and conventional drugs.

## 5. Conclusions

This study presents a promising approach for enhancing cancer therapy through the combination of AgNPs and MOFs, for the co-delivery of chemotherapeutic agents such as Cis-Pt. Evidence suggests a synergistic relationship between UiO-66-NH_2_ and AgNPs or Cis-Pt; however, further investigation is necessary to elucidate the precise mechanisms underlying these effects. Also, the preliminary data shows that UiO-66-NH_2_@AgNPs@Cis-Pt enhances the cytotoxicity of both AgNPs and Cis-Pt. However, the absence of a significant escalation in toxicity in comparison to UiO-66-NH_2_@AgNPs and UiO-66-NH_2_@Cis-Pt necessitates further investigation into the mechanisms of action of this nano-construct. The study of release mechanisms, the assessment of the cell cycle, apoptosis, and the identification of potential DNA repair pathways that could mitigate the combined damage induced by AgNPs and Cis-Pt are needed. Despite this, our preliminary findings underscore the potential of using MOF-based nanocarriers as effective tools for the delivery of AgNPs and chemotherapeutic agents in combination therapies. With further optimization, this approach has the potential to provide a basis for the development of more effective and targeted cancer therapies.

## Figures and Tables

**Table 1:** BET surface areas and ICP-OES quantification in total weight % of all the material synthetised.

	BET Surface Area	wt% Ag	wt% Pt
UiO-66-NH_2_	1037 m^2^/g	-	-
UiO-66-NH_2_@AgNPs	805 m^2^/g	0.19 wt%	-
UiO-66-NH_2_@Cis-Pt	931 m^2^/g	-	1.59 wt%
UiO-66-NH_2_@AgNPs@Cis-Pt	504 m^2^/g	0.20 wt%	6.50 wt%

## Data Availability

The original contributions presented in this study are included in the article and Supplementary Material and indicated as representative data. The raw data supporting the conclusions of this article will be made available by the authors on request, directed to the corresponding authors.

## Supplementary Materials

The following supporting information can be downloaded at: https://www.mdpi.com/article/10.3390/pharmaceutics17040512/s1, Figure S1: EDX spectrum indicating the peaks of Zr and Ag of UiO-66-NH_2_@AgNPs. Figure S2: Energy-dispersive X-ray (EDX) spectrum of UiO-66-NH_2_@Cis-Pt. Figure S3: N2 adsorption isotherms of: UiO-66-NH_2_ as synthetized (black cubes), UiO-66-NH_2_ decorated with AgNPs performed at 77 K. Figure S4: N2 adsorption isotherms of: UiO-66-NH_2_ loaded with Cis-Pt and UiO-66-NH_2_ with both AgNPs and Cis-Pt at 77 K. Figure S5: DLS measurements of the UiO-66-NH_2_ (0.5 mg mL^−1^, water). Figure S6: DLS measurements of UiO-66-NH_2_@AgNPs (0.5 mg mL^−1^, water). Figure S7: DLS measurements of UiO-66-NH^2^@Cis-Pt (0.5 mg mL^−1^, water). Figure S8: DLS measurements of UiO-66-NH2@AgNPs@Cis-Pt (0.5 mg mL^−1^, water).

## Abbreviations

The following abbreviations are used in this manuscript:

NPs	Nanoparticles
AgNPs	Silver nanoparticles
MOFs	Metal-organic frameworks
UiO-66	Universitetet i Oslo-66
EPR	Enhanced permeation and retention
ROS	Reactive oxygen species
BET	Brunauer–Emmett–Telle
SEM	Scanning electron microscopes
PXRD	Powder X-ray diffraction
MTT	Thiazolyl blue tetrazolium bromide
