# Supplementary material for: Silver Nanoparticles Decorated UiO-66-NH2 Metal-Organic Framework for Combination Therapy in Cancer Treatment
Source: Pharmaceutics. 2025 Apr 13;17(4):512. doi: 10.3390/pharmaceutics17040512 (PMC12030114; doi:10.3390/pharmaceutics17040512)
Supplement: Supplementary file 1 [file pharmaceutics-17-00512-s001.zip › pharmaceutics-3534436-supplementary.pdf]

Supporting Information

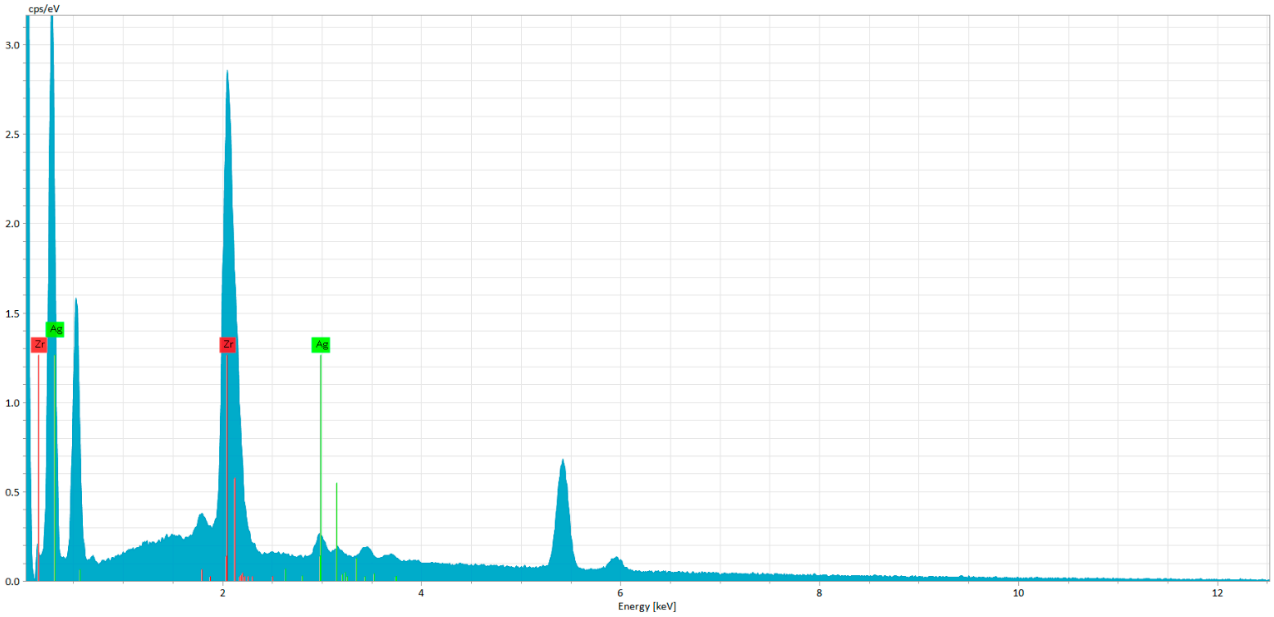

Figure S1: EDX spectrum indicating the peaks of Zr and Ag of UiO-66-NH_2@AgNPs.

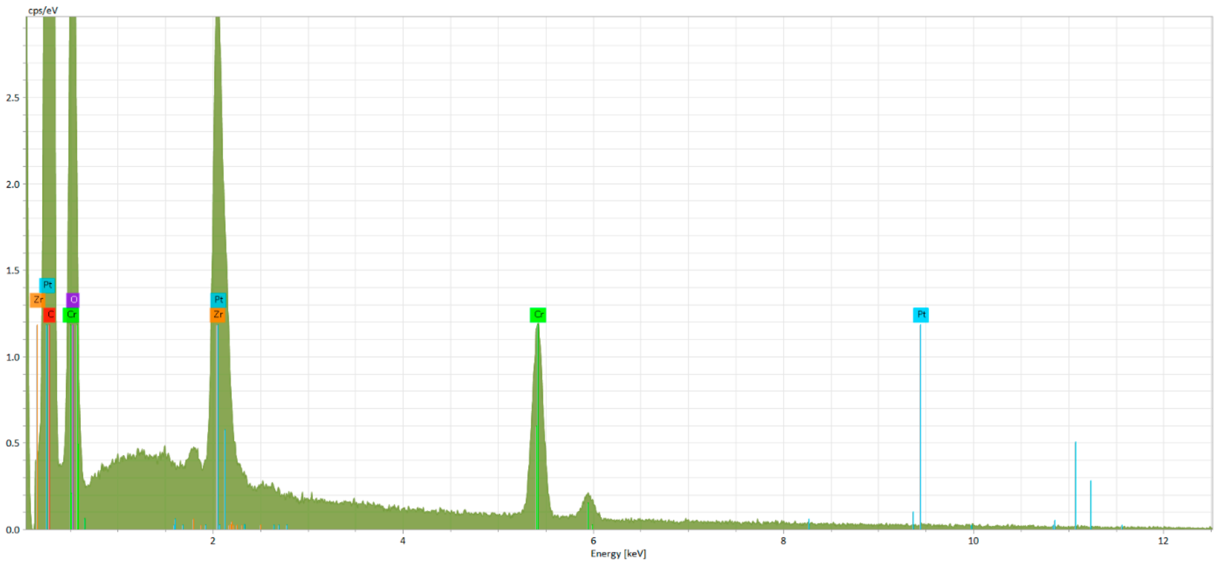

Figure S2: Energy-dispersive X-ray (EDX) spectrum of UiO-66-NH_2@Cis-Pt.

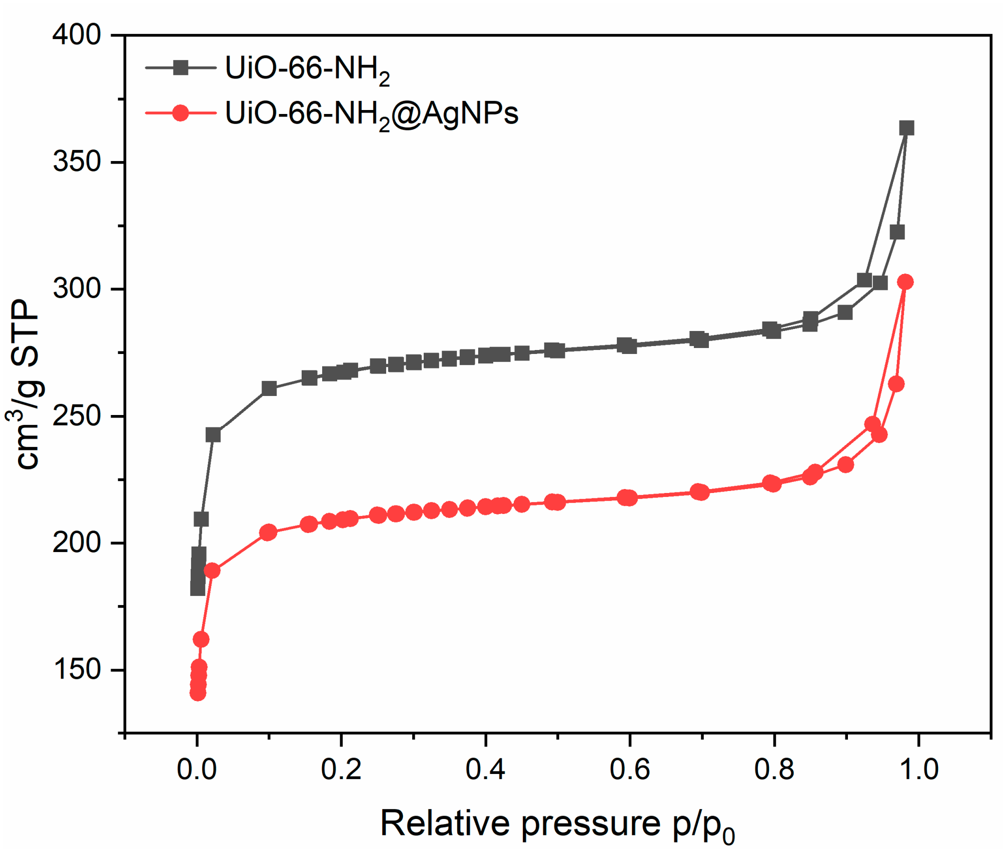

Figure S3: N_2 adsorption isotherms of: UiO-66-NH_2 as synthetized (black cubes), UiO-66-NH_2 decorated with AgNPs (red circles). The analysis was performed at 77 K.

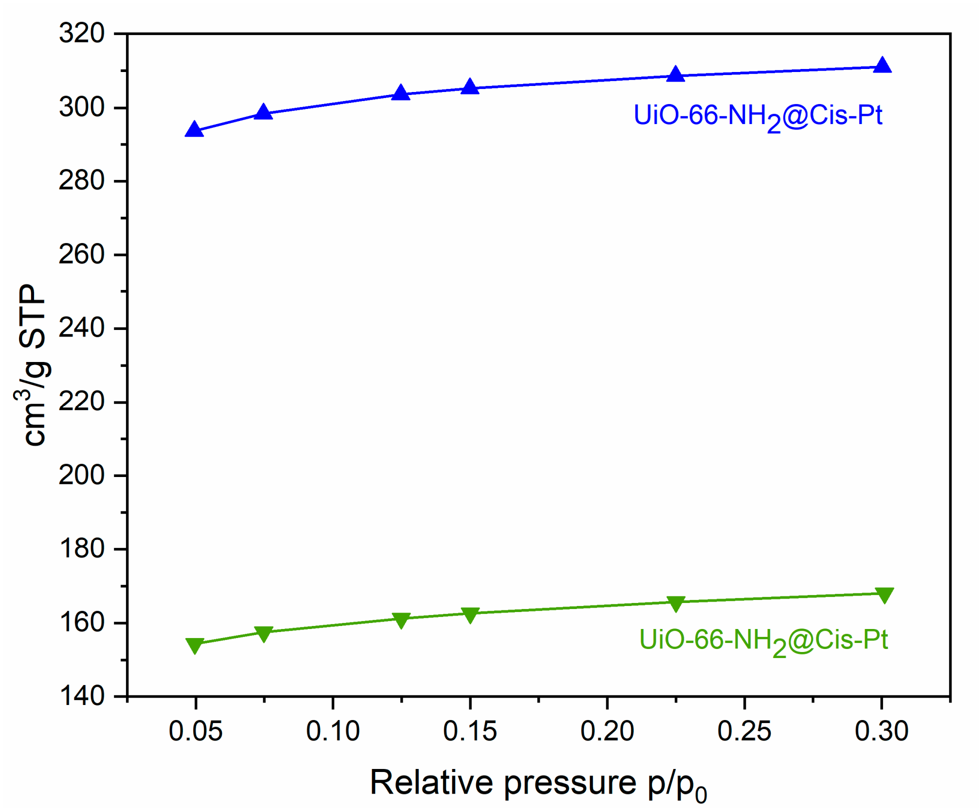

Figure S4: N_2 adsorption isotherms of: UiO-66-NH_2 loaded with Cis-Pt (blue triangles) and UiO-66-NH_2 with both AgNPs and Cis-Pt. The analysis was performed at 77 K.

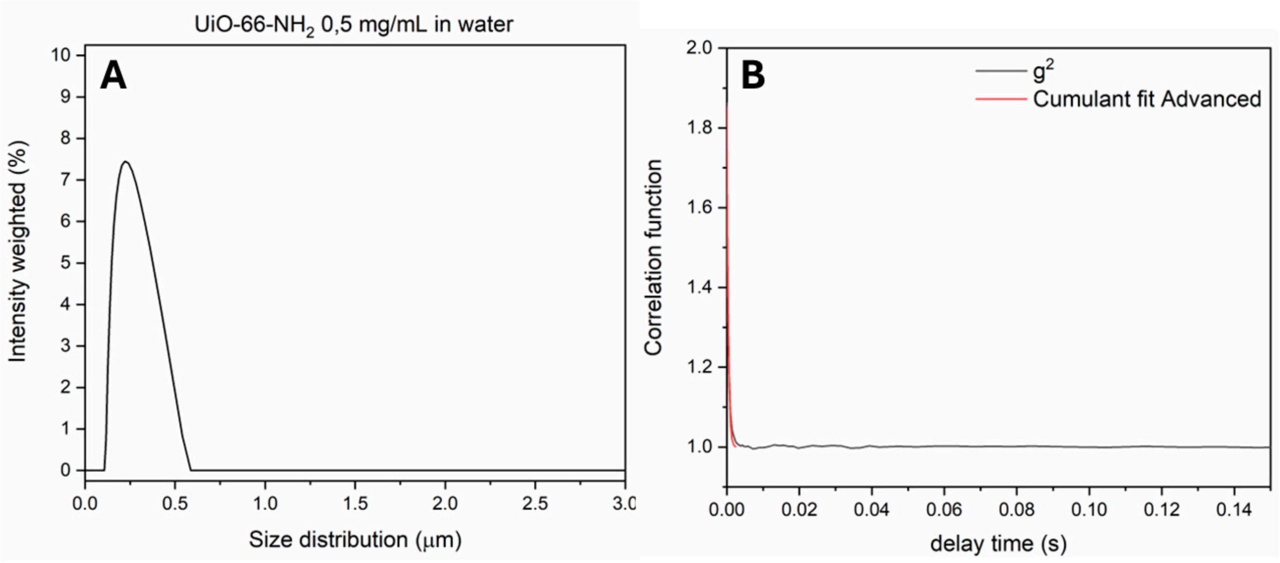

Figure S5: DLS measurements of the UiO-66-NH_2 (0.5 mg mL^{-1}, water)

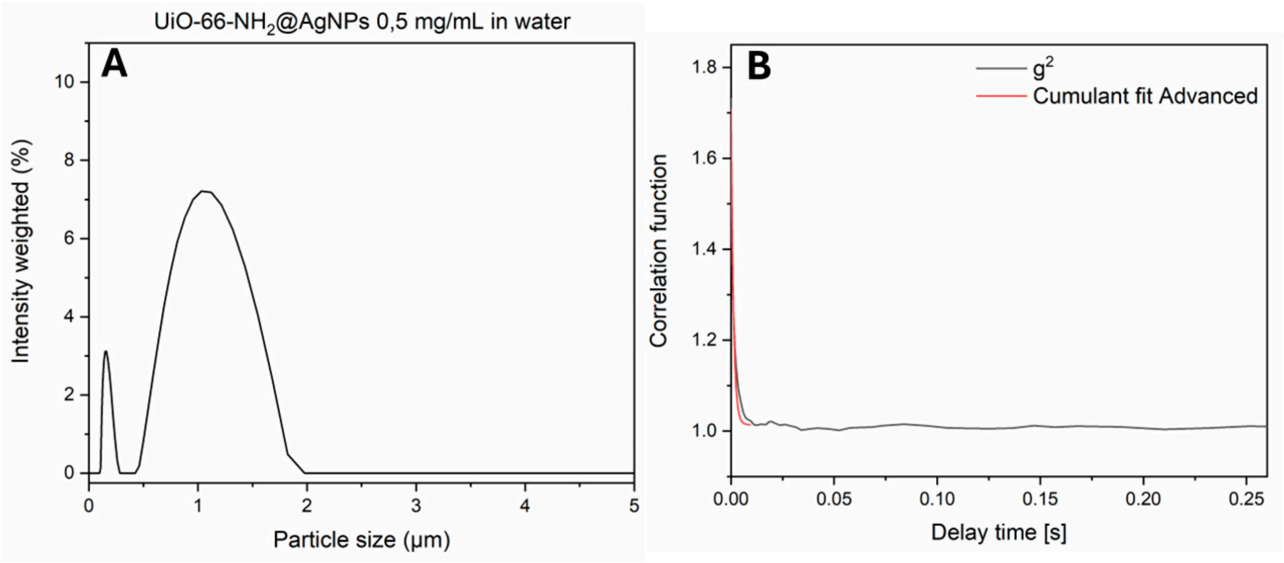

Figure S6: DLS measurements of UiO-66-NH_2@AgNPs (0.5 mg mL^{-1}, water)

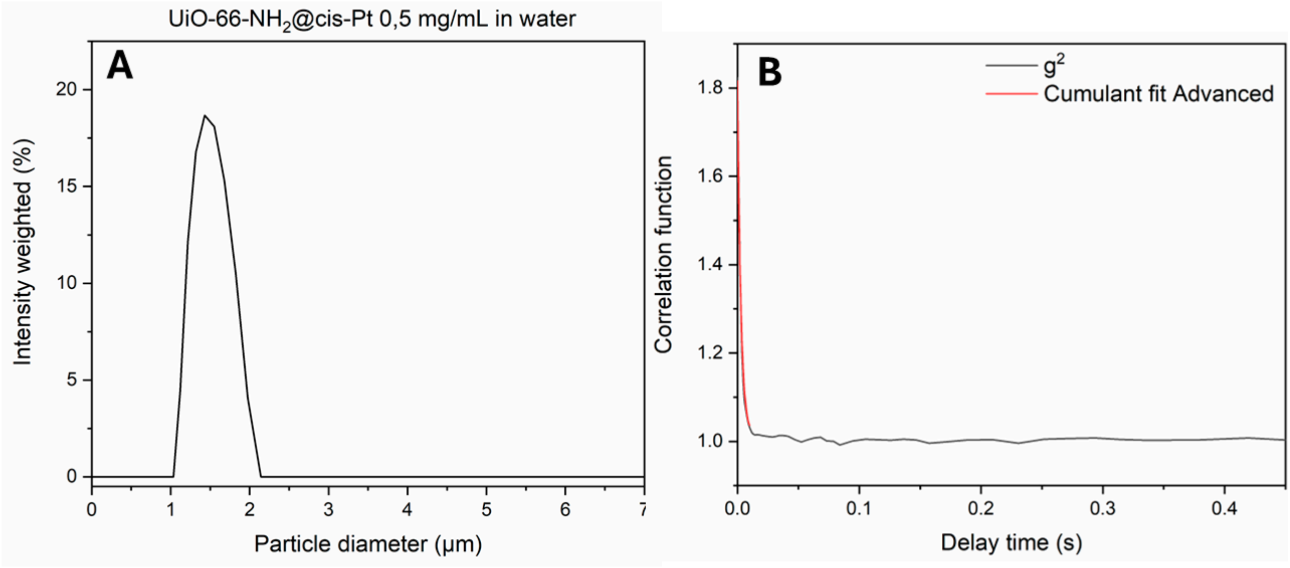

Figure S7: DLS measurements of UiO-66-NH_2@Cis-Pt (0.5 mg mL^{-1}, water)

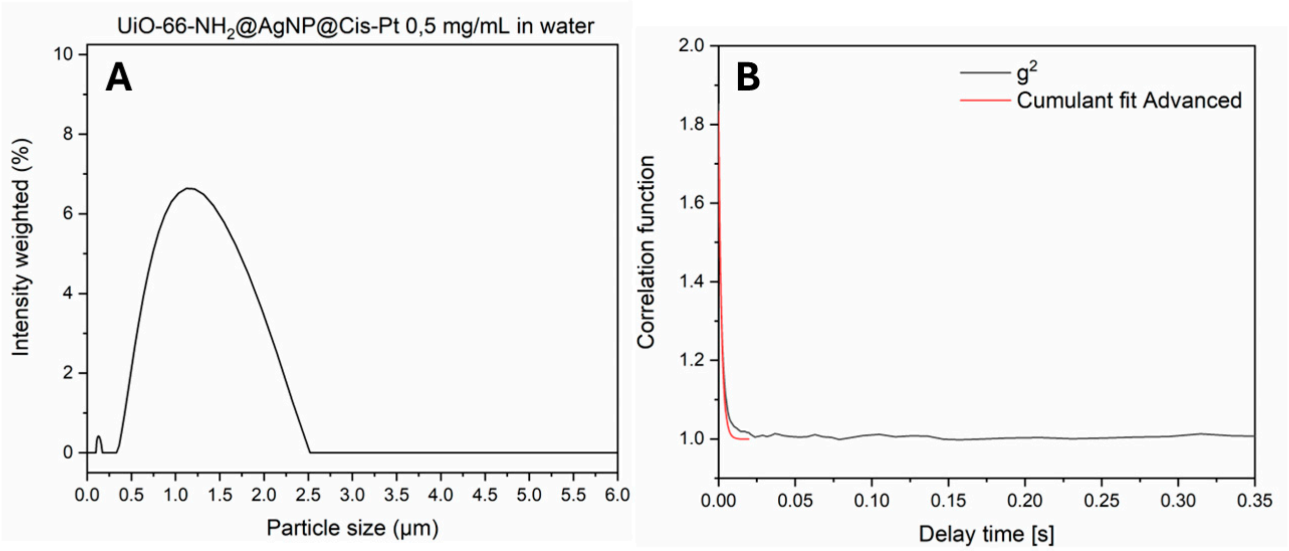

Figure S8: DLS measurements of UiO-66-NH_2@AgNPs@Cis-Pt (0.5 mg mL^{-1}, water)
